# Psychological determinants of health-related quality of life in primary care patients with diabetic foot ulcers: a **cross-sectional study in Singapore**

**DOI:** 10.3399/BJGPO.2025.0091

**Published:** 2025-12-19

**Authors:** Jiayi Weng, Xiaoli Zhu, Eng Sing Lee, Frederick HF Chan, Phoebe XH Lim, Ling Jia Goh, Jacqueline Giovanna De Roza, Yee Chui Chen, Konstadina Griva

**Affiliations:** 1 Khoo Teck Puat Hospital, Yishun Central, Singapore; 2 National Healthcare Group Polyclinics, Novena, Singapore; 3 Lee Kong Chian School of Medicine, Nanyang Technological University, Singapore

**Keywords:** diabetic foot, diabetes distress, illness perceptions, primary health care, quality of life, type 1 diabetes, type 2 diabetes

## Abstract

**Background:**

Diabetic foot ulcers (DFUs) significantly impair health-related quality of life (HRQoL). While clinical predictors are well established, the contribution of psychological factors, particularly in primary care, remains underexplored.

**Aim:**

To examine the sociodemographic, clinical, and psychological determinants of HRQoL in individuals with DFUs to inform development of psychologically informed interventions.

**Design & setting:**

Cross-sectional study conducted between April and October 2022 in primary care settings in Singapore.

**Method:**

A total of 186 patients with DFUs completed validated measures, including psychological variables (for example, the Diabetes Distress Scale and Brief Illness Perception Questionnaire) and Wound-QoL, which uses a questionnaire to assess quality of life in body, psyche, and everyday life domains. Hierarchical multiple regression analyses evaluated the contribution of psychological variables to HRQoL.

**Results:**

Psychological burden dominated, with psyche HRQoL impaired in 57% of participants (mean = 2.0), outpacing everyday life (38%; mean = 1.3) and body domains (24%; mean = 0.8). In hierarchical models, psychological variables — together with sociodemographic and clinical factors — explained 29.8% of the variance in body HRQoL, with interpersonal distress and threat perceptions emerging as significant predictors. A similar model accounted for 31.8% of the variance in psyche HRQoL, with female sex, emotional burden, and threat perceptions as key predictors. Everyday life HRQoL was significantly associated with HbA1c, independence in daily activities, emotional burden, and threat perceptions, with the full model explaining 33.7% of the variance.

**Conclusion:**

Psychological factors significantly contributed to reduced HRQoL in primary care patients with DFUs. Routine screening and targeted, psychologically informed support — particularly for females, those with poor glycaemic control, or limited functional independence — are essential to improve outcomes.

## How this fits in

While clinical predictors of health-related quality of life (HRQoL) in patients with diabetic foot ulcers (DFUs) are well-established, the role of psychological factors, especially in primary care, remains underexplored. This study identifies emotional burden, interpersonal distress, and threat-related illness perceptions as key psychological predictors of HRQoL, in addition to clinical and sociodemographic factors. The findings underscore the need for routine psychological screening and targeted, psychologically informed support — particularly for females, those with poor glycaemic control, and limited functional independence — to enhance outcomes and quality of life in primary care DFU management.

## Introduction

Diabetic foot ulcers (DFUs), affecting up to 34% of individuals with diabetes, represent one of the most serious complications of the disease, often resulting in infection and lower-limb amputation.^1^ Beyond these clinical sequelae, DFUs markedly impair HRQoL through chronic pain, reduced mobility, intensive wound care needs, and fears of recurrence or limb loss.^2–4^ Approximately 50% of individuals with DFUs report clinically significant depressive symptoms,^5^ underscoring the substantial psychological burden associated with the condition.

Despite this multidimensional impact, DFU management remains predominantly biomedical. While clinical factors, such as vascular disease, obesity, poor glycaemic control, and ulcer severity, are consistently linked to poorer HRQoL,^2,6–8^ psychological factors — particularly diabetes-related distress and illness perceptions — are increasingly recognised as critical yet underexplored determinants. Addressing these psychological dimensions is essential to advancing more holistic, patient-centred models of DFU care.

Diabetes distress reflects the psychological burden of managing diabetes and encompasses four domains: emotional burden, physician-related distress, regimen-related distress, and interpersonal distress. Elevated diabetes distress is associated with poorer HRQoL,^9,10^ suboptimal glycaemic control, and a higher risk of diabetes-related complications, including neuropathy.^11^ However, the mechanisms through which diabetes distress affects HRQoL in individuals with DFUs are not well understood.

Illness perceptions, shaped by sociocultural context, prior experiences, and health information, reflect individuals’ cognitive and emotional interpretations of their condition.^12^ These perceptions include dimensions such as identity (symptoms associated with the condition), perceived consequences (beliefs about its impact), timeline (perceived duration), personal control (belief in managing the illness), treatment control (beliefs in managing the illness through treatment), coherence (understanding the illness), emotional impact, and causal beliefs.^12–14^ Threat-related perceptions (for example, identity, consequences, timeline, concerns, and emotional responses) are linked to increased psychological distress, while control-related perceptions (for example, personal and treatment control, and coherence) are associated with better coping, treatment adherence, and improved HRQoL.^15–18^ Although illness perceptions have been shown to predict HRQoL and survival in hospital-based DFU cohorts,^19–21^ their relevance in primary care settings, where most DFU care occurs, remains underexplored.

HRQoL is a multifaceted, patient-centred construct reflecting individuals’ subjective evaluations of their physical, emotional, and social wellbeing.^22,23^ In the context of DFUs, psychological distress and negative illness beliefs are associated with reduced treatment adherence and prolonged healing,^24,25^ elevating risks of serious complications. However, the distinct and combined contributions of diabetes-related distress and illness perceptions to HRQoL in primary care populations with DFUs have yet to be systematically examined.

Understanding the psychological determinants of HRQoL in this population is essential, as poor HRQoL can impair self-care and increase the risk of amputation and mortality.^26,27^ Addressing these psychosocial factors within primary care could inform integrated care models that support both physical and emotional needs.

To address this gap, the present study employs the Wound-QoL, a validated, condition-specific instrument for assessing HRQoL in individuals with chronic wounds, including DFUs.^28^ The study aims to: 1) evaluate HRQoL in primary care patients with DFUs; 2) examine associations between HRQoL and sociodemographic, clinical, and psychological variables; and 3) determine the unique contributions of diabetes distress and illness perceptions to HRQoL, adjusting for relevant covariates.

## Method

### Study design and setting

This cross-sectional study was conducted between April and October 2022 in public primary care polyclinics in Singapore. Participants were recruited from nurse-led wound care services.

### Participants and recruitment

Eligible participants were adults (aged ≥21 years) with type 1 or type 2 diabetes and at least one active DFU, with the largest wound assessed. Exclusion criteria included inability to provide informed consent owing to cognitive impairment or severe psychiatric illness.

Surveys were self-administered in English, with optional assistance from trained research assistants fluent in English and Mandarin for participants with limited English proficiency. Research assistants received standardised training to provide consistent, culturally sensitive, and non-directive support, minimising response bias.

### Sample size

According to Faul *et al*,^29^ a sample size of approximately 184 participants is required to detect a small-to-medium effect size (*f*² = 0.05) with *α* = 0.05% and 80% power in a multiple regression analysis involving 20 predictors, as estimated using G*Power (version 3.1).

### Measures

HRQoL was assessed using the Wound-QoL,^28^ a 17-item measure with three subscales — body, psyche, and everyday life — that evaluate physical symptoms (for example, pain and odour), psychological burden (for example, worries), and daily functioning (for example, limitations on leisure activities). Items are rated on a five-point Likert scale (0 = not at all, 1 = a little, 2 = moderately, 3 = quite a lot, and 4 = very much), with higher scores indicating poorer HRQoL. A global score and three subscale scores are calculated by averaging item responses.

Diabetes distress was assessed using the 17-item Diabetes Distress Scale (DDS-17), which evaluates distress across four domains: emotional burden, physician-related distress, regimen-related distress, and interpersonal distress. Each item is rated on a six-point Likert scale, with scores of ≥3 indicating moderate to high levels of distress.^30^

Illness perceptions were assessed using the Brief Illness Perception Questionnaire, which evaluates cognitive and emotional representations of illness across eight dimensions.^13^ Based on established and empirical frameworks,^12,15–18^ items were grouped into threat perceptions (identity, consequences, timeline, concerns, and emotional beliefs) and control perceptions (personal control, treatment control, and illness coherence). Higher threat scores reflect more negative views of the illness; higher control scores indicate greater perceived manageability and understanding.

Sociodemographic and clinical covariates included age, sex, ethnicity, education level, housing type, activities of daily living (ADL) status, duration of diabetes, most recent HbA1c value, duration of DFU, ulcer recurrence (first versus recurrent), history of lower-limb amputation, and ulcer size.

### Statistical analysis

Guided by prior theoretical and empirical work,^15–17^ hierarchical multiple regression analyses were conducted to assess the extent to which HRQoL could be explained by independent variables entered in three sequential blocks. Block 1 included sociodemographic and clinical variables; block 2 comprised components of diabetes distress: emotional burden, physician-related distress, regimen-related distress, and interpersonal distress; and block 3 included illness perceptions, specifically threat and control perceptions. Durbin–Watson statistics for the three HRQoL subdomains ranged from 1.713–1.828, indicating no autocorrelation among residuals.

Descriptive statistics were used to summarise sample characteristics and study variables. Separate hierarchical regressions were conducted for each Wound-QoL subdomain (body, psyche, and everyday life), with changes in *R*² and *F*-statistics used to assess the explanatory value of each block. Incremental contributions of psychological variables were evaluated using changes in explained variance (Δ*R*²) and associated *F*-change statistics, allowing for identification of their unique influence beyond sociodemographic and clinical predictors.

All regression assumptions — linearity, normality, homoscedasticity, and absence of multicollinearity — were evaluated and satisfied. Multicollinearity was examined using tolerance values and variance inflation factor (VIF), with values <0.1 or >10 flagged as potential concerns. Missing data were assessed for randomness and addressed using pairwise deletion to optimise data retention while minimising bias. Sensitivity analyses were conducted as needed to confirm the robustness of the findings.

All analyses were conducted using SPSS Statistics (version 28), with statistical significance set at *P*<0.05 (two-tailed).

This manuscript was prepared in accordance with the Strengthening the Reporting of Observational Studies in Epidemiology guidelines.^31^

## Results

### Sample characteristics

The sample comprised 186 patients. Sociodemographic and clinical characteristics are presented in Table 1.

**Table 1.** Sociodemographic and clinical characteristics (*N* = 186)

Sociodemographic and clinical variables	*n*	%
Age, years		
<70	133	71.5
≥70	53	28.5
Sex		
Male	137	73.7
Female	49	26.3
Ethnicity		
Chinese	99	53.2
Non-Chinese	87	46.8
Education level		
Below secondary	85	45.7
Secondary and above	101	54.3
Employment		
Working	62	33.3
Not working	124	66.7
Dwelling		
1–3 room flat and rented	70	37.6
4–5 room flat, condominium, and landed property	116	62.4
Duration of diabetes		
<5 years	14	7.5
≥5 years	172	92.5
Duration of DFU		
<3 months	58	31.2
≥3 months	128	68.8
First DFU		
No	93	50.0
Yes	93	50.0
History of amputation		
No	102	54.8
Yes	84	45.2
ADL		
Require assistance	35	18.8
Independent	151	81.2
HbA1c		
≤7	51	27.4
>7	135	72.6
Number of DFU		
1	116	62.4
>1	70	37.6
Wound size		
<10 cm^2^	162	87.1
≥10 cm^2^	24	12.9

ADL = activities of daily living. DFU = diabetic foot ulcer.

### Patients reported outcome measures (PROMs)

**Figure 1.**
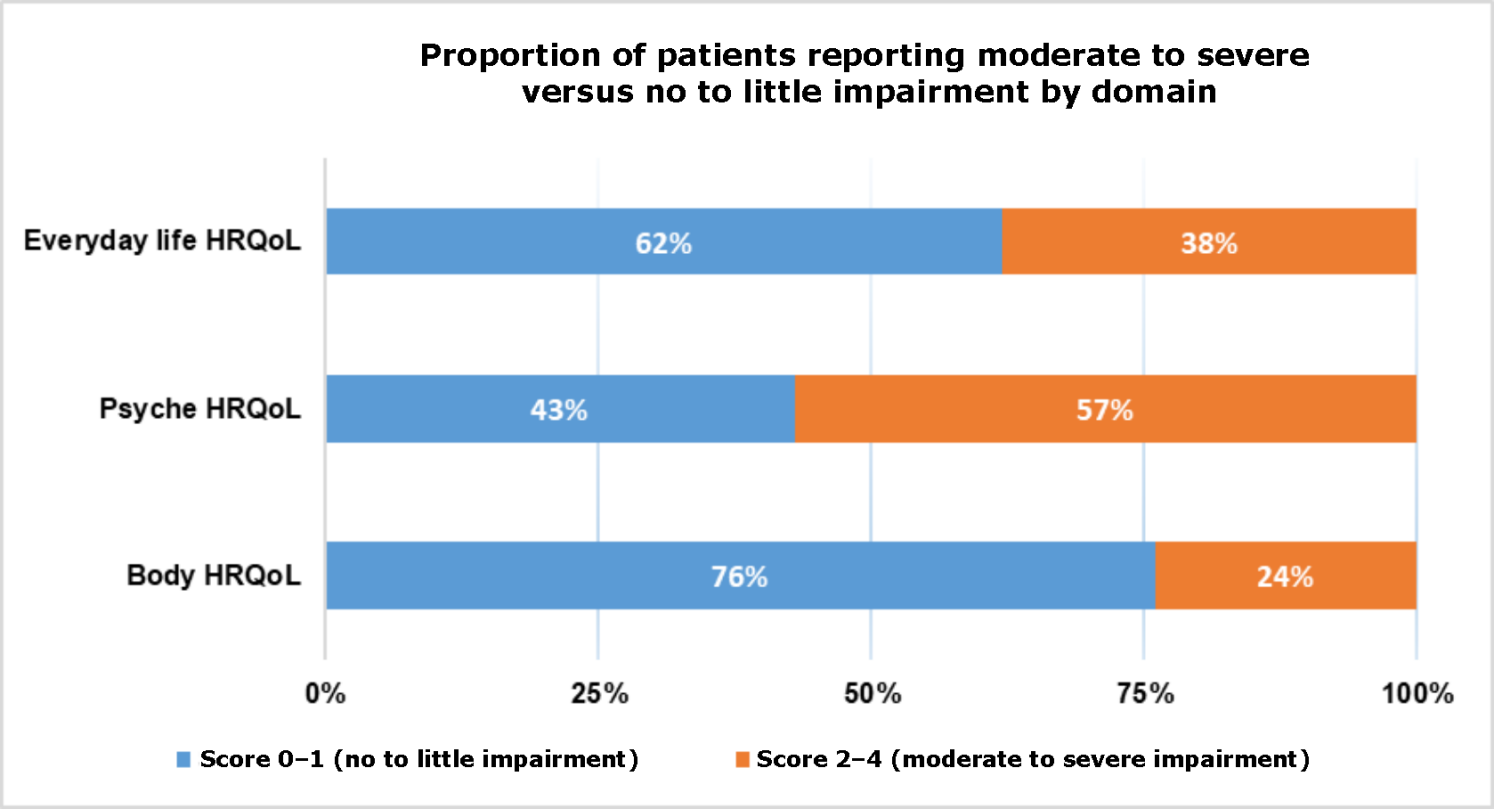
The proportion of participants reporting health-related quality of life (HRQoL) impairments by domain

Average DDS scores across the four domains ranged from 1.7–2.6 (standard deviation [SD] 0.9–1.3; scale 1–6), with ’emotional burden’ scoring highest, indicating moderate diabetes-related psychological distress. Mean threat perception was 25.5 (SD 11.4; range 0–50) and mean control perception was 21.4 (SD 6.5; range 0–30). The mean global Wound-QoL score was 1.4 (SD 0.9; range 0–4). Among subdomains, the psyche subscale (mean = 2.0, SD 1.2) was highest, reflecting moderate emotional burden, followed by everyday life (mean = 1.3, SD 1.2) and body (mean = 0.8, SD 0.9), indicating milder impacts on daily functioning and physical concerns (data not shown). Overall, 57% of patients reported moderate-to-severe impairment in psyche HRQoL (score 2–4), compared with 38% in everyday life and 24% in body (Figure 1).

These findings underscore the pronounced psychological impact of DFUs relative to other aspects of HRQoL.

### Hierarchical regression analyses of factors associated with HRQoL

Hierarchical regression analyses revealed that psychological factors — especially emotional burden and threat-related illness perceptions — consistently and significantly improved the prediction of HRQoL across all domains, beyond sociodemographic and clinical variables.

#### Body Wound-QoL

In Model 1, no sociodemographic or clinical variables significantly predicted body HRQoL (adjusted *R*² *=* 0.028, *F* change [degrees of freedom {df} 14, 171] = 1.377, *P* = 0.169).

Model 2 showed a substantial improvement (adjusted *R*² *=* 0.256, Δ*R*² = 0.228, *F* change [df 4, 167] = 14.139, *P*<0.001), with emotional burden, regimen-related distress, and interpersonal distress emerging as significant predictors.

In Model 3**,** the inclusion of illness perceptions explained an additional 4.5% of the variance (Δ*R*² = 0.045, *P*<0.01), bringing the total explained variance to 29.8%. In this final model, threat perceptions and interpersonal distress remained significant predictors (*F* change [df 2, 165] = 5.934, *P* = 0.003).

#### Psyche Wound-QoL

Model 1 explained only 1.3% of the variance (adjusted *R*² *=* 0.013, *F* change [df 14, 171] = 1.171, *P* = 0.302), with female sex as the only relevant factor.

Model 2 significantly increased the explained variance to 24.9% (Δ*R*² = 0.235, *P*<0.001), with emotional burden and female sex as significant predictors (*F* change [df 4, 167] = 14.437, *P*<0.001).

Model 3 added illness perceptions, contributing an additional 7% of the variance (Δ*R*² = 0.070, *P*<0.001). The final model explained 31.8% of the total variance in psyche HRQoL (adjusted R² = 0.318). In the final model, emotional burden, threat perceptions, and female sex remained significant predictors (*F* change [df 2, 165] = 9.473, *P*<0.001).

#### Everyday life Wound-QoL

Model 1 accounted for 14.7% of the variance (adjusted *R*² *=* 0.147, *F* change [df 14, 171] = 3.269, *P*<0.001), with elevated HbA1c and greater independence in ADLs as significant predictors.

Model 2 added diabetes distress, increasing the explained variance to 26.8% (Δ*R*² = 0.128, *P*<0.001), with emotional burden emerging as an additional significant predictor (*F* change [df 4, 167] = 8.094, *P*<0.001).

Model 3 included illness perceptions, which contributed an additional 7% (Δ*R*² = 0.070, *P*<0.001), resulting in a final model (adjusted *R*² *=* 0.337) where HbA1c, independent ADL, emotional burden, and threat perceptions were significant (*F* change [df 2, 165] = 9.711, *P*<0.001). This final model explained 33.7% of the total variance in Everyday Life HRQoL.

Across all domains, control-related illness perceptions (personal control, treatment control, and perceived coherence) showed no significant association with HRQoL. Tables 2 [Table table3] [Table table4]-5 present the summary of hierarchical regression models predicting HRQoL (Wound-QoL subdomains).

**Table 2.** Hierarchical linear regression analysis of demographic and clinical factors, diabetes distress, and illness perceptions by wound HRQoL dimensions: Model 1 (demographic and clinical variables)

	Body				Psyche				Everyday life			
	**Unstandardised coefficients**		**Standardised coefficients**		**Unstandardised coefficients**		**Standardised coefficients**		**Unstandardised coefficients**		**Standardised coefficients**	
**Category**	**B**	**Std error**	**Beta**	***P***	**B**	**Std error**	**Beta**	***P***	**B**	**Std error**	**Beta**	***P***
**Model 1**												
**Age (ref <70 years**)												
≥70 years	0.143	0.160	0.073	0.373	0.165	0.223	0.061	0.459	0.232	0.202	0.088	0.254
**Sex (ref male**)												
Female	0.009	0.156	0.005	0.952	0.455	0.217	0.164	**0.037**	-0.066	0.197	-0.025	0.736
**Ethnicity (ref Chinese**)												
Non-Chinese	-0.036	0.147	-0.020	0.809	-0.264	0.205	-0.108	0.199	-0.288	0.186	-0.121	0.123
**Education (ref below secondary**)												
Secondary above	-0.047	0.146	-0.026	0.749	0.151	0.203	0.062	0.456	0.152	0.184	0.064	0.409
**Employment (ref working**)												
Not working	-0.002	0.151	-0.001	0.988	0.041	0.210	0.016	0.847	0.043	0.191	0.017	0.823
**Dwelling (ref 1–3 room flat and below**)												
4–5 room flat above	0.090	0.141	0.049	0.523	-0.027	0.196	-0.011	0.892	-0.286	0.178	-0.116	0.110
**Duration of DM (ref <5 years**)												
≥5 years	-0.316	0.255	-0.094	0.216	-0.136	0.354	-0.029	0.703	-0.568	0.322	-0.126	0.079
**Duration of DFU (ref <3 months**)												
≥3 months	-0.289	0.149	-0.151	0.053	-0.212	0.206	-0.080	0.306	-0.287	0.187	-0.112	0.127
**First DFU (ref No**)												
Yes	-0.055	0.147	-0.031	0.706	-0.075	0.204	-0.031	0.712	-0.26	0.185	-0.109	0.162
**History of amputation (ref No**)												
Yes	-0.280	0.148	-0.158	0.060	-0.280	0.206	-0.114	0.175	-0.098	0.187	-0.041	0.600
**ADL (ref require assistance**)												
Independent	-0.303	0.180	-0.134	0.093	-0.181	0.250	-0.058	0.469	-0.800	0.227	-0.263	**<0**.**001**
**HbA1c (ref ≤7**)												
>7	0.056	0.150	0.028	0.708	-0.037	0.209	-0.014	0.858	0.471	0.189	0.176	**0.014**
**Number of DFU (ref 1**)												
>1	0.128	0.137	0.070	0.354	0.147	0.191	0.058	0.443	0.257	0.173	0.104	0.140
**Wound size (ref <10 cm^2^**)												
≥10 cm^2^	0.122	0.197	0.046	0.537	0.272	0.274	0.075	0.322	0.282	0.249	0.079	0.259
*R* ^2^ (adjusted *R* ^2^)		0.101 (0.028)			0.087 (0.013)				0.211 (0.147)			
*F* for change in *R* ^2^		1.377			1.171				3.269			

ADL = activities of daily living. B = unstandardised regression coefficients. Beta = standardised regression coefficients. Bold = statistical significance. DFU = diabetic foot ulcer. DM = diabetes mellitus. HRQoL = health-related quality of life. Std = standard.

**Table 3. table3:** Hierarchical linear regression analysis of demographic and clinical factors, diabetes distress and illness perceptions by wound HRQoL dimensions: Model 2 (demographic and clinical variables, and DDS domains)

	Body				Psyche				Everyday life			
	**Unstandardised coefficients**		**Standardised coefficients**		**Unstandardised coefficients**		**Standardised coefficients**		**Unstandardised coefficients**		**Standardised coefficients**	
**Category**	**B**	**Std error**	**Beta**	***P***	**B**	**Std error**	**Beta**	***P***	**B**	**Std error**	**Beta**	***P***
**Model 2**												
**Age (ref <70 years**)												
≥70 years	0.194	0.142	0.099	0.174	0.178	0.197	0.066	0.369	0.249	0.190	0.094	0.191
**Sex (ref male**)												
Female	0.119	0.139	0.059	0.392	0.460	0.193	0.166	**0.018**	-0.045	0.186	-0.017	0.808
**Ethnicity (ref Chinese**)												
Non-Chinese	0.050	0.131	0.028	0.704	-0.090	0.182	-0.037	0.620	-0.172	0.175	-0.072	0.329
**Education (ref below secondary**)												
Secondary above	0.011	0.128	0.006	0.929	0.182	0.178	0.074	0.308	0.178	0.171	0.074	0.301
**Employment (ref working**)												
Not working	-0.008	0.135	-0.004	0.951	0.158	0.187	0.061	0.400	0.120	0.180	0.047	0.507
**Dwelling (ref. 1–3 room flat and below**)												
4–5 room flat above	0.170	0.126	0.093	0.180	0.194	0.174	0.077	0.268	-0.135	0.168	-0.055	0.423
**Duration of DM (ref <5 years**)												
≥5 years	0.025	0.234	0.007	0.915	0.176	0.324	0.038	0.586	-0.317	0.312	-0.070	0.311
**Duration of DFU (ref <3 months**)												
≥3 months	-0.166	0.132	-0.087	0.208	-0.064	0.182	-0.024	0.725	-0.179	0.176	-0.070	0.309
**First DFU (ref No**)												
Yes	0.006	0.129	0.004	0.961	0.003	0.179	0.001	0.987	-0.197	0.173	-0.083	0.257
**History of amputation (ref No**)												
Yes	-0.187	0.134	-0.105	0.164	-0.300	0.185	-0.122	0.107	-0.101	0.179	-0.042	0.572
**ADL (ref require assistance**)												
Independent	-0.212	0.159	-0.094	0.184	-0.025	0.221	-0.008	0.909	-0.691	0.213	-0.227	**0.001**
**HbA1c (ref ≤7**)												
>7	-0.083	0.133	-0.042	0.534	-0.176	0.184	-0.064	0.341	0.360	0.178	0.135	**0.044**
**Number of DFU (ref 1**)												
>1	0.074	0.120	0.040	0.542	0.059	0.167	0.023	0.724	0.195	0.161	0.079	0.228
**Wound size (ref <10 cm^2^**)												
≥10 cm^2^	0.151	0.173	0.057	0.382	0.322	0.239	0.088	0.180	0.315	0.231	0.089	0.174
DDSEMO	0.133	0.050	0.202	**0.009**	0.393	0.070	0.431	**<0**.**001**	0.259	0.067	0.291	**<0**.**001**
DDSPHY	0.075	0.078	0.080	0.342	0.002	0.109	0.001	0.989	0.014	0.105	0.011	0.891
DDSREGI	0.175	0.085	0.180	**0.040**	0.183	0.117	0.137	0.120	0.164	0.113	0.126	0.148
DDSINTE	0.186	0.064	0.223	**0.004**	0.003	0.088	0.003	0.970	0.018	0.085	0.016	0.830
*R* ^2^ (adjusted *R* ^2^)		0.329 (0.256)			0.322 (0.249)				0.339 (0.268)			
*F* for change in *R* ^2^		14.139			14.437				8.094			

ADL = activities of daily living. B = unstandardised regression coefficients. Beta = standardised regression coefficients. Bold = statistical significance. DDS = Diabetes Distress Scale. DDSEMO = Diabetes Distress Scale Emotional Burden. DDSINTE = Diabetes Distress Scale Interpersonal Distress. DDSPHY = Diabetes Distress Scale Physician-related Distress. DDSREGI = Diabetes Distress Scale Regimen-related Distress. DFU = diabetic foot ulcer. DM = diabetes mellitus. HRQoL = health-related quality of life. Std = standard.

**Table 4. table4:** Hierarchical linear regression analysis of demographic and clinical factors, diabetes distress, and IPs by wound HRQoL dimensions: Model 3 (demographic and clinical variables, DDS domains, and threat and control IP)

	Body				Psyche				Everyday life			
	**Unstandardised coefficients**		**Standardised coefficients**		**Unstandardised coefficients**		**Standardised coefficients**		**Unstandardised coefficients**		**Standardised coefficients**	
**Category**	**B**	**Std error**	**Beta**	***P***	**B**	**Std error**	**Beta**	***P***	**B**	**Std error**	**Beta**	***P***
**Model 3**												
**Age (ref <70 years**)												
≥70 years	0.205	0.141	0.105	0.148	0.167	0.192	0.062	0.387	0.209	0.185	0.079	0.261
**Sex (ref male**)												
Female	0.079	0.136	0.039	0.561	0.403	0.185	0.145	**0.031**	-0.086	0.178	-0.032	0.629
**Ethnicity (ref Chinese**)												
Non-Chinese	0.092	0.129	0.052	0.477	-0.038	0.176	-0.016	0.828	-0.144	0.169	-0.060	0.397
**Education (ref below secondary**)												
Secondary above	-0.090	0.128	-0.051	0.481	0.007	0.174	0.003	0.970	0.012	0.167	0.005	0.942
**Employment (ref working**)												
Not working	-0.036	0.132	-0.019	0.787	0.102	0.179	0.039	0.570	0.058	0.172	0.023	0.737
**Dwelling (ref 1–3 room flat and below**)												
4–5 room flat above	0.156	0.122	0.086	0.203	0.173	0.166	0.069	0.300	-0.153	0.160	-0.062	0.341
**Duration of DM (ref <5 years**)												
≥5 years	-0.003	0.227	-0.001	0.989	0.122	0.309	0.026	0.694	-0.375	0.297	-0.083	0.208
**Duration of DFU (ref <3 months**)												
≥3 months	-0.185	0.128	-0.097	0.150	-0.093	0.174	-0.035	0.595	-0.202	0.167	-0.079	0.228
**First DFU (ref No**)												
Yes	0.013	0.126	0.007	0.918	0.012	0.171	0.005	0.943	-0.190	0.164	-0.080	0.249
**History of amputation (ref No**)												
Yes	-0.197	0.130	-0.111	0.133	-0.308	0.177	-0.125	0.084	-0.099	0.170	-0.041	0.562
**ADL (ref require assistance**)												
Independent	-0.124	0.158	-0.055	0.434	0.143	0.215	0.046	0.506	-0.515	0.207	-0.169	**0.014**
**HbA1c (ref ≤7**)												
>7	-0.062	0.130	-0.031	0.632	-0.132	0.176	-0.048	0.453	0.409	0.169	0.153	**0.017**
**Number of DFU (ref 1**)												
>1	0.010	0.119	0.006	0.930	-0.057	0.161	-0.023	0.725	0.078	0.155	0.032	0.617
**Wound size (ref <10 cm^2^**)												
≥10 cm^2^	0.180	0.168	0.068	0.285	0.367	0.228	0.101	0.110	0.352	0.220	0.099	0.111
DDSEMO	0.064	0.053	0.098	0.225	0.277	0.072	0.304	**<0**.**001**	0.151	0.069	0.170	**0.030**
DDSPHY	0.057	0.077	0.061	0.462	-0.038	0.104	-0.030	0.715	-0.032	0.100	-0.025	0.752
DDSREGI	0.142	0.083	0.146	0.090	0.121	0.113	0.090	0.285	0.100	0.109	0.076	0.358
DDSINTE	0.200	0.062	0.240	**0.002**	0.023	0.085	0.020	0.783	0.032	0.082	0.029	0.693
Threat IP	0.021	0.006	0.267	**<0**.**001**	0.036	0.008	0.332	**<0**.**001**	0.034	0.008	0.322	**<0**.**001**
Control IP	-0.003	0.01	-0.020	0.774	0.005	0.013	0.026	0.702	0.015	0.013	0.078	0.245
*R* ^2^ (adjusted *R* ^2^)		0.374 (0.298)			0.392 (0.318)				0.409 (0.337)			
*F* for change in *R* ^2^		5.934			9.473				9.711			

ADL = activities of daily living. B = unstandardised regression coefficients. Beta = standardised regression coefficients. Bold = statistical significance. DDS = Diabetes Distress Scale. DDSEMO = Diabetes Distress Scale Emotional Burden. DDSINTE = Diabetes Distress Scale Interpersonal Distress. DDSPHY = Diabetes Distress Scale Physician-related Distress. DDSREGI = Diabetes Distress Scale Regimen-related Distress. DFU = diabetic foot ulcer. DM = diabetes mellitus. HRQoL = health-related quality of life. IP = illness perceptions. Std = standard.

**Table 5.** Summary of hierarchical regression models predicting HRQoL (Wound-QoL subdomains)

HRQoL domain	Model	Significant predictors	Δ*R*²	Adjusted *R*²	*F* change	*P*-value
**Body**	Model 1	None	–	0.028	1.377	0.169
	Model 2	Emotional burden, regimen distress, and interpersonal distress	0.228	0.256	14.139	<0.001
	Model 3	Threat perceptions and interpersonal distress	0.045	0.298	5.934	0.003
**Psyche**	Model 1	Female sex	–	0.013	1.171	0.302
	Model 2	Female sex and emotional burden	0.235	0.249	14.437	<0.001
	Model 3	Female sex, emotional burden, and threat perceptions	0.070	0.318	9.473	<0.001
**Everyday life**	Model 1	HbA1c and independent ADL	–	0.147	3.269	<0.001
	Model 2	HbA1c, independent ADL, and emotional burden	0.128	0.268	8.094	<0.001
	Model 3	HbA1c, independent ADL, emotional burden, and threat perceptions	0.070	0.337	9.711	<0.001

ADL = activities of daily living. HRQoL = health-related quality of life.

## Discussion

### Summary

This study highlights the significant impact of psychological factors — emotional burden, interpersonal distress, and threat-related illness perceptions — on HRQoL in individuals with DFUs, beyond sociodemographic and clinical variables, within primary care settings where wound management and shared decision making are central. These findings emphasise the need for patient-centred, psychologically informed care that includes routine psychological assessment and targeted interventions, particularly for high-risk groups such as females, individuals with poor glycaemic control, and those with ADL impairments.

### Strengths and limitations

A major strength of this study is the use of the Wound-QoL, a condition-specific PROM with strong content validity for diabetes-related foot disease.^32–34^ In the absence of a gold-standard instrument,^35^ the Wound-QoL’s needs-based approach prioritises patient experiences over clinical metrics,^36^ capturing wound-specific concerns such as pain, mobility, emotional distress, and functional impairment. Additionally, hierarchical multiple regression allowed systematic evaluation of the unique contribution of psychological variables to HRQoL beyond sociodemographic and clinical factors, highlighting the incremental predictive value of emotional burden and threat perceptions while adjusting for potential confounders.

Several limitations should be acknowledged. The cross-sectional design offers only a single time-point perspective, limiting causal inference, while reliance on self-reported data may introduce recall or social desirability biases. Although illness perceptions were grouped into ’threat’ and ’control’ domains based on established theoretical frameworks,^15–18^ qualitative research^37^ highlights that patient beliefs may diverge from biomedical models, potentially complicating communication with healthcare providers. Future research should employ longitudinal designs to clarify causal pathways and better capture psychosocial needs across the DFU care continuum. Incorporating patient-derived frameworks — through both quantitative and qualitative methodologies, including volition-based or culturally adapted constructs — may enhance the sensitivity and contextual relevance of psychosocial assessments.

Although social support was not directly assessed, the observed interpersonal distress and emotional burden suggest it may influence DFU outcomes. This is further supported by the link between reduced functional independence and poorer everyday life HRQoL. Future research should assess both the adequacy and types of social support to inform more personalised care.

Similarly, health literacy was not assessed, and the high proportion of participants without secondary education may have affected illness understanding and response accuracy. Including validated measures of health literacy and social determinants (for example, income, housing, and caregiving) would strengthen contextual insights and support equitable, patient-centred intervention development.

### Comparison with existing literature

This study confirmed several known predictors of HRQoL in individuals with DFUs. Female sex was significantly associated with poorer psychological HRQoL, suggesting higher emotional distress among females than males. This finding aligns with previous studies reporting poorer HRQoL in women with DFUs,^7,38,39^ particularly in psychological domains.^39^ These disparities may reflect differences in coping, social roles, or access to psychosocial resources, highlighting the need for gender-sensitive approaches.

Elevated HbA1c levels and reduced independence in ADL were significantly associated with poorer everyday life HRQoL, reflecting impaired daily functioning, autonomy, and social participation. These findings align with evidence that poor glycaemic control and functional limitations negatively impact HRQoL.^40,41^ Chronic hyperglycaemia delays wound healing and increases complication risk, while ADL impairments further limit autonomy and engagement.^3^ Interventions targeting glycaemic control and functional independence may enhance clinical outcomes and HRQoL.

Emotional burden and threat-related illness perceptions consistently predicted reduced HRQoL across psychological, functional, and physical domains. Emotional burden, a core component of diabetes distress, was linked to poorer psychological and functional outcomes, aligning with evidence that distress lowers functioning and wellbeing in diabetes populations.^10^ Interpersonal distress, reflecting insufficient social support, was associated with poorer physical HRQoL, consistent with broader diabetes findings.^42^ These results align with UK research on the psychological toll of diabetes-related foot complications^43^ and underscore the need for psychologically informed, person-centred DFU care in primary and community settings. Notably, this study extends existing knowledge by demonstrating domain-specific impacts of diabetes distress in the DFU population.

Threat-related illness perceptions — reflecting beliefs about the severity and emotional impact of the condition — were significantly associated with lower HRQoL. This supports theoretical models and prior evidence that negative illness beliefs contribute to maladaptive coping and worse outcomes.^15–17^ Notably, unlike a prior study,^16^ control-related perceptions (personal control, treatment control, and illness coherence) were not significantly linked to HRQoL in this sample. This suggests that among individuals with DFUs, threat perceptions may have a more immediate effect on wellbeing than control-related beliefs. Further research should explore these mechanisms to inform tailored psychological interventions.

### Implications for research and practice

This study underscores the need for routine psychological assessment in DFU management in primary care settings. Incorporating brief, validated screening tools and regular HRQoL assessments can identify psychosocial risks early and guide personalised care strategies. Targeted interventions — such as psychoeducation, motivational interviewing, and social support — should prioritise high-risk groups.

There is a need for scalable, integrated care models that address the psychological needs of individuals with DFUs. Embedding motivational interviewing^44^ into routine wound care may help align patient beliefs with clinical perspectives by eliciting personal narratives, fostering autonomy, and providing emotional support. This approach aligns with UK primary care priorities emphasising continuity and person-centredness, supported by evidence that patients value emotional support from podiatrists and prefer continuity with the same provider.^43^

Poor HRQoL — driven by emotional burden, illness beliefs, and reduced functional independence — can undermine patients’ engagement in wound care and delay treatment-seeking behaviour. Integrating psychological support into routine DFU care may enhance wellbeing, promote adherence, and support healing. This aligns with primary care principles, where continuity and sustained patient–practitioner relationships provide an ideal context for embedding psychosocial support into chronic wound management. Future studies should address implementation challenges, including interprofessional collaboration, effective communication, and clinical leadership.^45^

In conclusion, psychological factors — particularly emotional burden, interpersonal distress, and threat-oriented illness perceptions — are key contributors to diminished HRQoL in individuals with DFUs. Routine psychological screening and targeted psychosocial interventions, especially for females, individuals with poor glycaemic control, and those with limited functional independence, are essential for improving outcomes and delivering person-centred care in primary care settings.

## Data

The dataset relied on in this article is available from the corresponding author on reasonable request.
